# A Review of Mobile Mapping Systems: From Sensors to Applications

**DOI:** 10.3390/s22114262

**Published:** 2022-06-02

**Authors:** Mostafa Elhashash, Hessah Albanwan, Rongjun Qin

**Affiliations:** 1 Geospatial Data Analytics Lab, The Ohio State University, Columbus, OH 43210, USA; elhashash.3@osu.edu (M.E.); albanwan.1@osu.edu (H.A.); 2 Department of Electrical and Computer Engineering, The Ohio State University, Columbus, OH 43210, USA; 3 Department of Civil, Environmental and Geodetic Engineering, The Ohio State University, Columbus, OH 43210, USA; 4 Translational Data Analytics Institute, The Ohio State University, Columbus, OH 43210, USA

**Keywords:** mobile mapping, LiDAR, positioning

## Abstract

The evolution of mobile mapping systems (MMSs) has gained more attention in the past few decades. MMSs have been widely used to provide valuable assets in different applications. This has been facilitated by the wide availability of low-cost sensors, advances in computational resources, the maturity of mapping algorithms, and the need for accurate and on-demand geographic information system (GIS) data and digital maps. Many MMSs combine hybrid sensors to provide a more informative, robust, and stable solution by complementing each other. In this paper, we presented a comprehensive review of the modern MMSs by focusing on: (1) the types of sensors and platforms, discussing their capabilities and limitations and providing a comprehensive overview of recent MMS technologies available in the market; (2) highlighting the general workflow to process MMS data; (3) identifying different use cases of mobile mapping technology by reviewing some of the common applications; and (4) presenting a discussion on the benefits and challenges and sharing our views on potential research directions.

## 1. Introduction

The need for regularly updated and accurate geospatial data has grown exponentially in the last decades. Geospatial data serve as an important source for various applications, including, but not limited to: indoor and outdoor 3D modeling, generation of geographic information system (GIS) data, disaster response high-definition (HD) maps, and autonomous vehicles. This data collection has been made possible through continuous advances in mobile mapping systems (MMSs). MMS refers to an integrated system of mapping sensors mounted on a moving platform to provide the positioning of the platform while collecting geospatial data [1]. A typical MMS platform uses light detection and ranging (LiDAR) and/or high-resolution cameras as its primary sensors to acquire data for objects/areas of interest, integrated with sensor suites for positioning and georeferencing, such as the global navigation satellite system (GNSS) and inertial measurement unit (IMU). To perform accurate georeferencing, traditional mobile mapping approaches require extensive post-processing, such as strip adjustment of point cloud scans or bundle adjustment (BA) of images using ground control points (GCPs), during which manual operations may be required to clean noisy data and unsynchronized observations. Recent trends of MMSs have aimed to perform direct georeferencing and to leverage the capabilities of a multi-sensor platform [2,3] in order to minimize human interventions during data collection and processing. Automation has been further strengthened to use machine learning/artificial intelligence to perform online/offline object extraction and mappings, such as traffic lights and road sign extraction [4,5,6].

Mobile mapping technology has undergone significant development in the past few decades, with algorithmic advances in photogrammetry, computer vision, and robotics [7]. In addition, increased processing power and storage capacity have further facilitated the collection speed and data volume [8]. The applications and systems have been further strengthened by the availability of a diverse set of low-cost survey sensors with various specifications, making mobile mapping more flexible and able to acquire data in complex environments (e.g., tunnels, caves, and enclosed spaces) with lower cost and labor expenditures [9]. Typically, commercial MMSs can be classified (based on their hosting platforms) into handheld, backpack, trolley, and vehicle-based. Some platforms are designed to work indoors without relying on GNSS, while others can work indoors and outdoors. Mobile mapping technology gained more attention when it was adopted by companies such as Google and Apple [10,11] for various applications including navigation and virtual/augmented reality [12].

**Figure 1:**
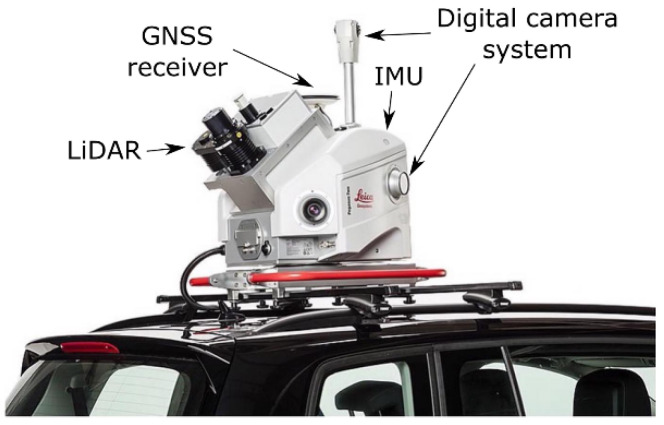
An example of an MMS: a vehicle-mounted mobile mapping platform consisting of different positioning and data collection sensors to generate an accurate georeferenced 3D map of the environment. Shown here are the main sensors of the Leica Pegasus: Two Ultimate as an example. Photo courtesy of Leica Geosystems [13].

An example of a vehicle-based MMS (Leica Pegasus: Two Ultimate [13]) and its typical sensor suites is shown in Figure 1. It consists of both data acquisition sensors and positioning sensors. The data acquisition sensors primarily consist of a calibrated LiDAR and digital camera suite. The LiDAR sensor is capable of producing 1,000,000 points/second and the camera suite captures a 360° horizontal field of view (FoV) to provide both texture/color information and stereo measurements, if needed. The positioning sensors include a GNSS receiver that provides the global positional information, with an additional IMU and distance measuring instrument (DMI) that obtains odometry information for integrated position correction. These positioning systems are required to be calibrated in their relative positions and play a vital role in the generation of globally-consistent point clouds.

Despite the existence of a few mobile mapping technologies in the market, the technology landscape of MMS is highly disparate. There is no single and standard MMS that is widely used in the mapping community. Most of the existing MMSs are customized using different sensor suites at different grades of integration. As such, each has its pros and cons. Previous studies have largely focused on comparative studies among certain devices [1,14,15,16,17,18,19] or targeted systems for specific application scenarios (e.g., indoors or outdoors) [20,21,22]. Due to the rapid development of imaging, LiDAR, positioning sensors, and onboard computers, the updated capabilities of these essential components of an MMS may not be fully reflected through a few integrated systems, rendering such studies less informative. There is a general dearth of studies covering the comprehensive landscape of sensor suites and respective MMSs. In this paper, we focused on providing a meta-review of sensors and platforms tasked for ground-level 3D mappings, as well as the techniques needed to integrate these sensors as suites for MMSs in different application scenarios. This review was intended to provide an update on sensors, MMSs with different hosting platforms, and the extended applications of MMSs. The aim was to serve not only researchers in the field of mobile mapping, with updated background information, but also practitioners on critical factors of concern when customizing an MMS for specific applications. We highlighted the main steps from data acquisition to refinement and discussed some of the most common challenges and considerations of MMS.

## 2. Paper Scope and Organization

This paper was intended to provide a comprehensive review of MMS technology, including a thorough discussion, covering mobile mapping from sensors and software to their applications. We thoroughly discussed the different types of sensors, their practical capabilities, and their limitations, as well as methods of fusing sensory data. We then described the main platforms that are currently used for mapping tasks in different application scenarios (e.g., indoor and outdoor applications). In addition, we discussed the main stages of processing MMS data, including preprocessing, calibration, and refinement. In order to assess the benefits of an MMS in practice, we examined a few of the most important applications that widely use mobile mapping technology. Finally, for the benefit of future work, we highlighted the main considerations and challenges in an open discussion.

The rest of this paper has been organized as follows: Section 3 provides a detailed review of essential positioning and data collection sensors in MMSs; Section 4 presents the different MMS hosting platforms, based on their application scenarios (i.e., vehicle-mounted systems, handheld, wearable, and trolley-based); Section 5 presents the workflow to process MMS data, from acquisition to algorithms for fusing their observations and refinement; Section 6 introduces the enabled applications using MMS for mapping and beyond. Finally, Section 7 concludes this review and discusses future trends.

## 3. An Overview of Sensors in Mobile Mapping Systems

Positioning and data collection sensors are two classes of essential components used in a typical MMS, as depicted in Figure 1. Positioning sensors are used to obtain the geographical positions and motion of the sensors, which are used to georeference the collected 3D data. Examples of these sensors include GNSS, IMU, DMI (i.e., odometers), etc. To achieve more statistically accurate positioning, the measurements from these sensors are usually jointly used through fusion. Addition fusion can also be performed between the position and navigation cameras. Sensor fusion solution for positioning is currently standard, as neither the GNSS receiver nor the IMU/DMI alone can provide sufficiently accurate and reliable measurements for navigating mobile platforms. GNSS measurements are usually subject to signal strength variation in different environments; for example, one could obtain a strong signal in open spaces and weak signal or signal loss in tunnels or indoors, leading to a loss of information. On the other hand, the IMU and DMI are subject to a significant accumulation of errors and are often used as supplemental observations for navigation when GPS data are available.

Data collection sensors mostly consist of LiDAR and digital cameras, providing raw 3D/2D measurements of the surrounding environments. The 3D measurements of an MMS rely on LiDAR sensors, while the images are primarily used to provide colorimetric/spectral information [20]. With the development of advanced dense image matching methods [23,24,25,26,27], these images are also collected stereoscopically to provide additional dense measurements for 3D data fusion. In the following subsections, we provided an overview of positioning and data collection sensors, as well as the respective sensor fusion approaches.

### 3.1. Positioning Sensors

As mentioned above, typical positioning sensors include GNSS receiver, IMU, and DMI. Their patterns regarding errors are complementary; thus, in modern MMS, they are often used, through a sensor fusion solution, to provide accurate positioning information up to the centimeter [28]. Nevertheless, their individual measurement accuracies are still critical to the accuracy of the resulting 3D maps. An overview of the positioning sensors is shown in Table 1. In the following subsections, we discussed the three main positioning sensors: GNSS, IMU, and DMI.

#### 3.1.1. Global Navigation Satellite System Receiver

The GNSS receiver is a primary source used to estimate absolute position, velocity, and elevation in open areas referenced to a global coordinate system (e.g., WGS84). It passively receives signals from a minimum of four different navigational satellite systems and performs trilateration to calculate its real-time positions. Since it depends on an external source of signal, the GNSS often exhibits fewer accumulation errors or none at all. These satellite systems mainly refer to the GPS developed by the United States, the GLONASS (Globalnaya Navigatsionnaya Sputnikovaya Sistema) developed by Russia, the Galileo built by the European Union, and the BeiDou system developed by China [29]. The raw observations (pseudo-range, carrier phase, doppler shifts, etc.) from the chipset of the receiver with its solver often give a positional error at the meter level, depending on the chipsets and antenna (e.g., single/dual frequencies) [30]. High-tier MMSs often use augmented GPS solutions, such as Differential GPS (DGPS) or Real-Time Kinematic GPS (RTK-GPS), to improve the positioning accuracy to decimeters and centimeters (and can achieve an accuracy of 1 cm [31]). DGPS uses a code-based measure and can operate with single-frequency receivers without initialization time, while RTK-GPS uses carrier-phase measures and requires dual-frequency receivers. The latter takes about one minute to initialize (for fixing wavenumbers) [19]. Both DGPS and RTK-GPS rely on a network of reference stations, linked to a surveyed point in its vicinity, to apply corrections and eliminate various errors such as ionosphere delays and other unmodeled errors. The traditional DGPS method achieves submeter accuracy in the horizontal position, while, with much more advanced techniques and solvers, the RTK-GPS, as a type of DGPS, can achieve centimeter-level accuracy in three dimensions. However, these achievable accuracy measures are conditioned to open areas; when collecting 3D data in dense urban areas with tall buildings or indoor environments, the GNSS signal can be heavily impacted by occlusions and the resulting measurements can be inaccurate [32]. As such, it requires other complimentary sensors when operating under such conditions. In general, the positioning platform of an MMS is expected to achieve an accuracy of 5–50 mm at speeds that can reach the maximum speed of highways (120–130 km/h) when considering the integration of complementary sensors.

#### 3.1.2. Inertial Measurement Unit

IMU is an egocentric sensor that records the relative position of the orientation and directional acceleration of the host platform. Its positional information can be calculated through dead reckoning approaches [33,34]. Unlike GNSS, it does not require links to external signal sources, and it records relative positions with respect to a reference to its starting point (which can usually be dynamically provided by GNSS in open fields). Like many other egocentric navigation methods, it suffers from accumulation errors, often leading to significant drifts to its true positions. To be more specific, an IMU consists of an accelerometer and a gyroscope which it uses to sense acceleration and angular velocity. These raw measurements are fed into an onboard computing unit to apply the dead reckoning algorithm to provide real-time positioning. Thus, the IMU and computing unit, together with the algorithm as a whole, are also called an inertial navigation system (INS). The grade/quality of IMU sensors can be differentiated by the type of gyroscope: a majority of light-weight, consumer-grade IMUs use microelectromechanical systems (MEMS), which are affordable but suffer from poor precision and large drift errors (often 10–100°/h [35]) [3,36]. Higher grade systems for precise navigation use a larger but more accurate gyroscope, e.g., a ring laser or fiber optic gyroscope, which can reach a drift error of less than 1°/h [35]. IMU can work in GPS-denied environments indoors, outdoors, and in tunnels. However, given its use of dead reckoning navigation, its measurements will only be accurate for a relatively short period in reference to the starting point. Since GNSS provides reasonable accuracy in an open area and its measurements do not have error accumulations as the platform moves, it often integrates IMU for additional observations. This, as a standard approach, provides more accurate positional information in complex environments mixed with both open and occluded surroundings [37].

#### 3.1.3. Distance Measuring Instrument

The DMI generally refers to instruments that measure the traveled distance of the platform. In many cases, DMI is alternatively referred to as the odometer or wheel sensor for MMS based on vehicles or bikes. It computes the distance based on the number of cycles the wheel rotates. Since DMI only measures distance, it is often used as supplementary information to GNSS/IMU as an effective means to reduce the accumulated errors and constrain the drift from IMU in GPS-denied environments such as tunnels [38]. It requires calibration before use and measures distance, velocity, and acceleration.

### 3.2. Sensors for Data Collection

Data collection sensors are another major component in an MMS, used to collect 3D data. They typically refer to sensors such as LiDAR and high-resolution cameras that provide both geometry and texture information. They require constant georeferencing using the position and orientation information provided by the positioning sensors to link the 3D data to the world coordinate system. In this section, we introduced LiDAR and imaging systems (i.e., cameras), described their functions, types, benefits, challenges, and limitations, and provided an example of a system representing the status quo.

#### 3.2.1. Light Detection and Ranging (LiDAR)

LiDAR, or light detection and ranging, is an optical instrument that uses directional laser beams to measure the distances and locations of objects. It provides individual and accurate point measurement on a 3D object; thus, many of these measurements together constitute information about the shape and surface characteristics of objects in the scene. It has many desirable features in a 3D model, as it is highly accurate, can acquire dense 3D information in a short time, exhibits invariance to illumination, and can partially penetrate sparse objects like canopies. LiDAR itself is still an instrument used for measuring relative locations. It requires a suite of highly accurate and well-calibrated navigation systems to retrieve global 3D points, the installation and cost of which, in addition to the already expensive LiDAR sensor, make it a high-cost means of collection.

The concept of using light beams for distance measurements has existed since 1930 [39]. Since the invention of the laser in 1960, LiDAR technology has experienced rapid development [40] and has been very popular for accurate mapping and autonomous driving applications [41]. Nowadays, there are many commercially-available LiDAR sensors for surveying or automotive applications. Typically, survey-grade LiDAR achieves a range accuracy at the millimeter level (usually 10–80 mm); examples include the RIEGL VQ-250, VQ-450 [42], and Trimble MX9 and MX50 [43]. Relatively lower-grade LiDAR sensors (which are also lower in cost) achieve a range accuracy at the centimeter level (usually 1–8 cm), generally satisfying applications for obstacle avoidance and object detection. These are often used in autonomous driving platforms given their good tradeoff between cost and performance. Examples of such LiDAR sensors include Velodyne [44], Ouster [45], Luminar Technology [46], and Innoviz Technologies [47]. In fact, the level of accuracy of different grades of LiDAR sensors and their costs are the main deciding factors when considering the choice of a LiDAR sensor. There is a large cost gap between grades, with survey-grade LiDARs often costing hundreds of thousands of USD (at the time of this publication) and relatively lower-grade ones coming in approximately ten times cheaper.

LiDAR sensors can be categorized, based on their collecting principles, into three main categories: rotating, solid-state, and flash. Rotating LiDAR uses a rotating mirror spinning for 360 degrees and redirecting laser beams. It usually has multiple beams, and each beam illuminates one point at a time. The rotating LiDAR is the most commonly used in MMS; based on its rotating nature, it provides large FoV, high signal-to-noise ratio, and dense point clouds [48]. Solid-state LiDAR usually uses MEMS mirrors, embedded in a chip [49] through which the mirror can be controlled to follow a specific trajectory or optical phased arrays to steer beams [50]. As such, it is considered solid because it does not possess any moving parts in the sensor. Flash LiDAR [51] usually illuminates the entire FoV with a wide beam in a single pulse. Analogous to a camera with a flash, a flash LiDAR uses a 2D array of photodiodes to capture the laser returns, which are finally processed to form 3D point clouds [48,52]. Typically, a Flash LiDAR has a limited range (less than 100 m) as well as a limited FoV, constrained by the sensor size. Although LiDAR is primarily used to generate point clouds, it can also be used for localization purposes through different techniques such as scan matching [53,54,55]. The extractable information can be further enhanced by deep neural networks for semantic segmentation and localization [56,57]. However, like many other methods, while LiDAR sensors can provide relatively accurate range measurements, their performance deteriorates significantly in hazardous weather conditions such as heavy rain, snow, and fog.

Table 2 shows an example of several existing LiDAR sensors along with their technical specifications in terms of range, accuracy, number of beams, FoV, resolution, point per second, and refresh rate. Generally, the choice of sensors depends on the application and the characteristics of the moving platform (e.g., the speed, payload, etc.). As mentioned above, most MMSs rely on rotating LiDAR sensors, but they often come at a high cost compared to other categories. Therefore, using solid-state LiDAR in an MMS is a promising direction, since its cost is lower than rotating LiDAR. When the vehicle speed is high, there is less time to acquire data, and more beams are needed to ensure that the object of interest is measured by sufficient points [44,58]. For instance, a 32-beam LiDAR could be sufficient for a vehicle moving at a speed of 50–60 km/h, but a LiDAR with 128 beams is recommended for higher speeds, up to 100–110 km/h, so that the acquired data have an adequate resolution. The operating range of a LiDAR can be also important and should be considered on an application basis (e.g., long-range LiDAR may be unnecessary for indoor applications.). In general, the cost usually increases by a factor of 1.5–2 when the number of beams is doubled; this is also positively correlated to the operating range.

#### 3.2.2. Imaging Systems and Cameras

Imaging systems like cameras are among the most popular sensors used for data collection due to their low-cost and ability to provide high-resolution texture information. Cameras are usually mounted on the top or front of the moving platform to capture information about the surrounding environment. They are intended to acquire many images at a high frame rate i.e., 30–60 frames per second. Cameras are useful to serve a few main purposes. First, they are used for recovering the geometry of the scene, usually obtained through stereoscopic/binocular cameras that process a pair of overlapping images and recover depth information using stereo-dense image matching approaches [24,25,26]. Second, they are capable of obtaining the textures of objects in the scene (a camera records photons of the object at different spectral frequencies that provide rich and critical information about the object’s natural appearance) and can be used to build panoramic and geotagged images, as well as photorealistic models. Third, the texture information gathered by cameras encodes critical semantics of the object and can be used to detect static objects such as traffic lights, stop signs, markings, and road lanes. They can also detect moving objects such as pedestrians and cars, which is gradually becoming more applicable as modern deep learning methods are developed to tackle such problems [4,59,60].

There are many types of camera sensors and configurations used in MMSs, depending on their intended use, as described earlier. Examples include monocular cameras, binocular cameras, RGB-D cameras, multi-camera systems (e.g., ladybug), fisheye, etc. A summary of different camera types is shown in Table 3. Monocular cameras (low-cost cameras) provide a series of single RGB images without any additional depth information and are often used to collect high-resolution and geotagged images or panoramas [11]. However, they cannot be used to recover 3D scale or generate high accurate 3D points. Binocular cameras, on the other hand, consist of two cameras capturing synchronized stereo images to recover depth and scale, with an additional computational cost incurred through stereo-dense image matching techniques [24,25,26]. The performance and accuracy of the 3D information depend on the selection of the stereo-dense image matching method. In many cases, mapping solutions may rely on RGB-D cameras (e.g., Kinect [61], Intel RealSense D435 [62]) which can provide both RGB images and depth images (through structured light) simultaneous. They are primarily used in indoor settings due to their limited range. Integrating LiDAR with RGB-D images can yield highly accurate 3D information; however, this may require precompensation for the uncertainties in the RGB-D images from random noise or occlusions. Due to the compact/cluttered environment, an MMS often includes a wide FoV or even 360° panoramic camera, which is usually achieved via a multi-camera system that uses a group of synchronized cameras sharing the same optical center (e.g., FLIR Ladybug5+ [63]). Panoramic images additionally facilitate the integration with LiDAR scanners, providing 100% overlap between the image and LiDAR point clouds (e.g., those from rotating LiDAR). As a result, the panoramic images are suitable for street mapping applications. As a lower-cost alternative, a fisheye camera aims to provide an image with extended FoV from a single camera. It has a spherical lens that can provide more than 180° FOV. Although this is cheaper, the savings may come at the cost of image distortions in scale, geometry, shape, and illumination, requiring additional and slightly more complex calibrations. 

As a modern MMS benefits from various cameras providing additional information, it comes with a few added complexities. First, it captures images using the reflected light off of objects, which makes it sensitive to the illumination of the environment, such as the high dynamic range of the scene (between sky and ground) and hazy weather conditions [64,65]. Second, cameras and multi-camera systems require calibration to reduce different types of distortions [66]. Third, moving platforms require high framerate cameras to leverage the speed, image quality, and resolution [67].

## 4. Mobile Mapping Systems and Platforms

There are a few factors that determine the type of sensor and platform to be used for MMS tasks. These factors include available sensors, project budget, technical solutions, processing strategies, and scene contents (i.e., indoor or outdoor). These help to determine the type of available sensors (e.g., with/without GPS) and the accessible platforms (e.g., vehicle-mounted or backpack, etc.). For example, in indoor environments, there is no access to GPS signals or vehicles, thus, alternative solutions must be adopted.

In general, we considered broadly categorizing the MMS platforms into traditional vehicles and nontraditional lightweight/portable mapping devices. Traditional vehicle-based MMSs primarily operate on main roads, collecting city or block-level 3D data. The nontraditional portable devices, such as backpack/wearable systems, handheld systems, or trolley-based systems, depending on their application and task, can be used both outdoors or indoors in GPS-denied environments. For outdoor applications, these means of mapping are primarily used to complement vehicle-based systems, mapping narrow streets, and areas that cannot be accessed by larger vehicles [68,69]. For indoor or GPS-denied environments, the sensor suites may be significantly different from those used outdoors; for example, they may primarily rely on INS or visual odometry for positioning [70,71]. To be more specific, in this section, we introduced four typical MMS platforms that offered mapping solutions, namely a traditional vehicle platform and three portable platforms (handheld, wearable, and trolley-based systems). Further details of these systems are provided in Table 4 and the following subsections.

### 4.1. Vehicle-Mounted Systems

**Figure 2:**
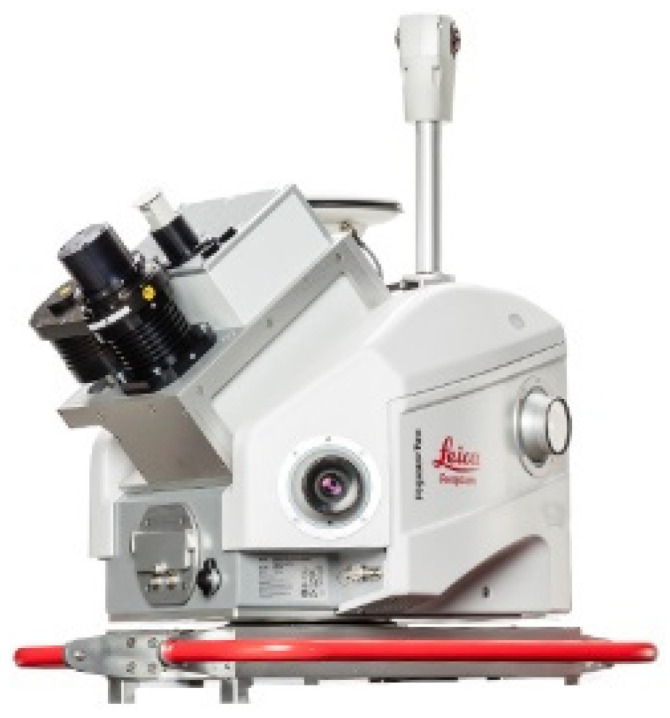
Leica Pegasus: Two Ultimate vehicle-mounted system. Photo courtesy of Leica Geosystems [13].

This setting refers to mounting the sensor suites on top of a vehicle to capture dense point clouds. These systems enable a high rate of data acquisition at the vehicle travel speed (20–70 mph). The sensor platform can be mounted on cars, trains, or boats, depending on the mapping application. Generally, vehicle-mounted systems achieve the highest accuracy compared to other mobile mapping platforms, primarily because of their size and payload, which allows them to host high-grade sensors [72]. A vehicle-mounted MMS system is usually equipped with a survey-grade LiDAR that provides dense and accurate measurements, as well as a deeply integrated 360° FoV camera providing textural information. Regarding the positioning sensors, a vehicle-mounted system usually fuses measurements from GNSS receivers with IMU and DMI. An example of these systems is introduced in [73], where one Velodyne HDL-32E, and five Velodyne VLP-16 LiDAR sensors were combined with multiple GPS receivers and IMUs. Other examples include Leica Pegasus: Two Ultimate [13], Teledyne Optech Lynx HS600-D [74], Topcon IP-S3 HD1 [75], Hi-Target HiScan-C [76], Trimble MX50, MX9, MX7 [43], and Viametris vMS3D [77]. Figure 2 shows a sample of such vehicle-mounted systems.

Vehicle-mounted systems are used for various applications, such as urban 3D modeling, road asset management, and condition assessment [78,79]. Moreover, these systems can be used for automated change detection in the mapped regions [80,81], creating up-to-date HD maps as an asset for autonomous driving [73] and railway monitoring applications [82].

Although vehicle-mounted systems play a major role in mobile mapping, their relatively large size hinders their accessibility to many sites, such as narrow alleys and indoor environments. Additionally, some studies [83] have demonstrated that the speed of the vehicle may affect the quality of the 3D data, creating doppler effects over successive scans [84]. Therefore, the speed and route have to be planned ahead of the mapping mission.

### 4.2. Handheld and Wearable Systems

**Figure 3:**
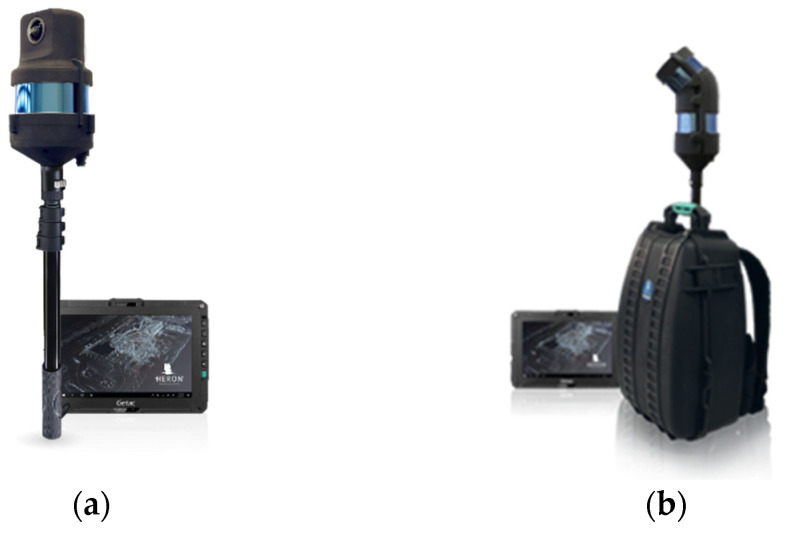
Handheld and wearable systems: (**a**) HERON LITE Color, (**b**) HERON MS Twin. Photos courtesy of Gexcel srl [87].

The handheld and wearable systems follow lightweight and compact designs using small-sized sensors. An operator can hold or wear the platform and walk through the area of interest. Wearable systems are often designed as a backpack system to allow the operator to collect data while walking. Both handheld and wearable systems are distinguished by their portability, which enables mapping GPS-denied environments such as enclosed spaces, complex terrains, or narrow spaces that vehicles cannot access [20,85]. Due to the nature of these environments, handheld and wearable systems may not rely on GNSS receivers for positioning, but could instead depend on an IMU or use a LiDAR and camera for both data collection and localization (using simultaneous localization and mapping (SLAM) approaches) [86]. A sample of several handheld and wearable systems is shown in Figure 3. Examples of these devices include HERON LITE Color [87], GeoSLAM Zeb Revo Go, Zeb RT, Zeb Horizon [88], Leica BLK2GO [13], Leica Pegasus: Backpack [13], HERON MS Twin [87], NavVis VLX [89], and Viametris BMS3D-HD [77]. Some other examples of these devices are introduced in [70,85,90], where they showed the benefit of using LiDAR with IMU to generate 2D and 3D maps and evaluated the performance of these mapping devices in indoor environments.

As mentioned above, handheld and wearable systems are effective in mapping enclosed spaces; for instance, these devices can be used to map caves where GNSS signals and lighting are not available [91]. In addition, they are used to map cultural heritage sites that may be complex and require data to be efficiently collected from different viewing points [92,93,94]. Furthermore, these systems are efficient in mapping areas that are not machine-accessible, such as forest surveying [68,95,96], safety and security maps, and building information modeling (BIM) [97]. However, working in GPS-denied regions requires compensating for the lost signal, which demands setting GCPs in these regions, or utilizing GPS information before entering into such environments [98], whereas, inside tunnels, navigation completely depends on IMU/DMI or scanning sensors (LiDAR or cameras).

### 4.3. Trolley-Based Systems

**Figure 4:**
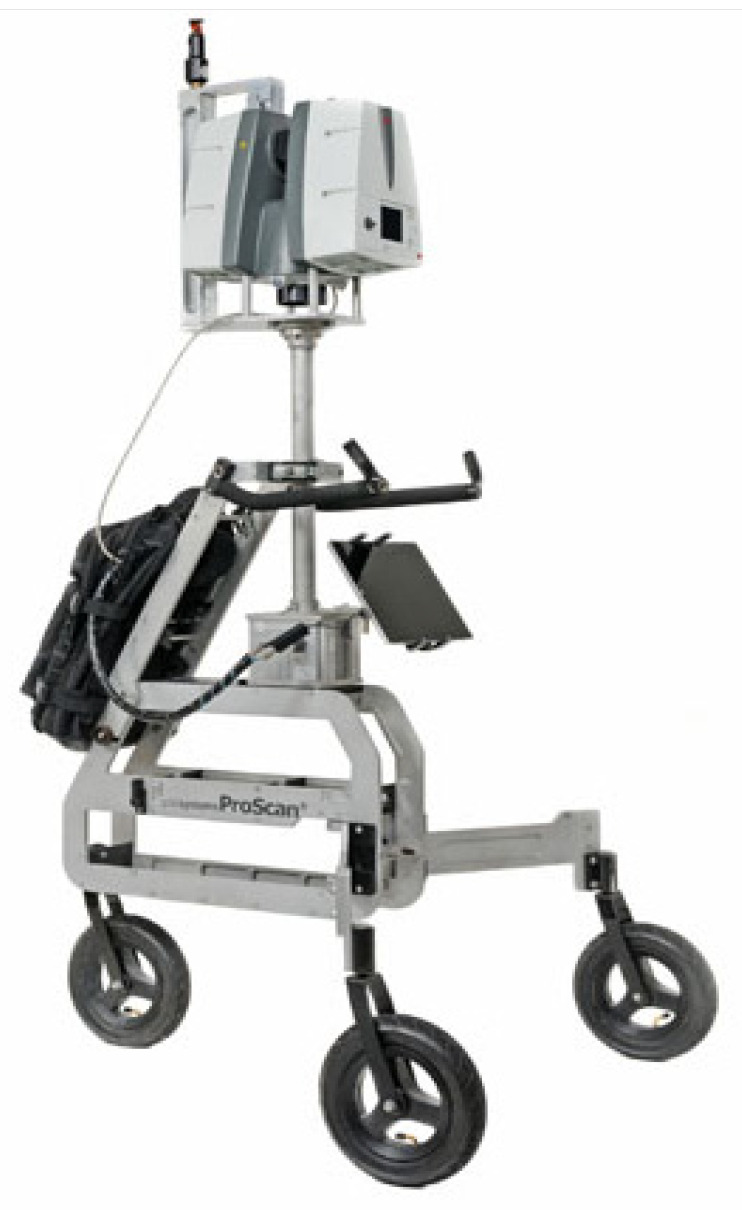
Leica ProScan trolley-based MMSs. Photo courtesy of Leica Geosystems [13].

This type of system is similar in nature to the backpack system while maintaining the ability to be slightly more sizeable and carry a heavier payload. It is suitable for indoor and outdoor mapping where the ground is flat [99]. A sample of trolley-based systems is shown in Figure 4. Examples of these systems include NavVis M6 [89], Leica ProScan [13], Trimble indoor [43], and FARO Focus Swift [100]. Trolley-based systems are also suitable for a variety of applications, such as tunnel inspection, measuring asphalt roughness, creating floorplans, and BIM [22]. In addition, they are used for creating 3D indoor geospatial views of all kinds of infrastructure, such as plant and factory facilities, residential and commercial buildings, airports, and train stations.

## 5. MMS Workflow and Processing Pipeline

**Figure 5:**
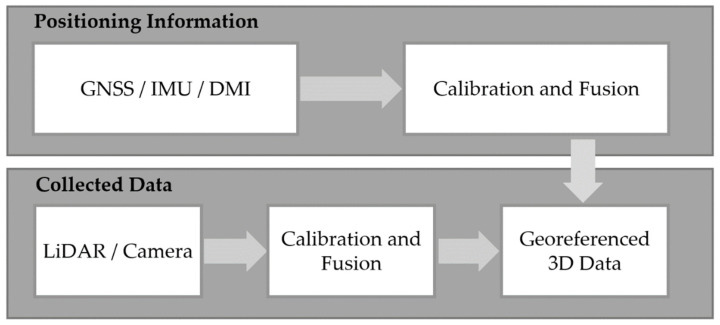
The standard processing pipeline for MMS data.

There are a few processing steps required to turn raw sensory data from an MMS to the final 3D product. These generally include data acquisition, sensor calibration and fusion, georeferencing, and data processing in preparation for scene understanding (shown in Figure 5). In the following subsections, we provided an overview of these typical processing steps.

### 5.1. Data Acquisition

The planned route must be analyzed to determine the configured platforms and sensors to be deployed; for example, the operators should be aware of the GNSS accessible regions in order to plan the primary sensors to use. For MMS positioning, the GNSS, IMU, and DMI continuously measure the position and motion of the platform. In most outdoor applications, the main navigation and positioning data are provided by the GNSS satellite to the receiver, and the IMU and DMI supplement measurements where GNSS signals are insufficient or lost. In some specific cases where GNSS is completely inaccessible, such as cave mapping, GCPs will be used to reference the data to the geodetic coordinate system. Additionally, 3D data are mainly collected by an integrated LiDAR and camera system, where LiDAR produces accurate 3D point clouds colorized by the images from the associated camera.

### 5.2. Sensors Calibration and Fusion

Sensor calibration and fusion are often performed throughout the data collection cycle. The goal of this is to calibrate the relative positions between multiple sensors, including between cameras, between camera and LiDAR, or among LiDAR, camera, and navigation sensors. Additionally, their output must be fused as a postprocessing step to achieve more accurate positional measurements. These serve multiple purposes: more accurate localization, more accurate geometric reconstruction, and data alignment for fusion [101,102]. In the following subsections, we introduced a few typical calibration procedures in MMS.

#### 5.2.1. Positioning Sensors Calibration and Fusion

The integration of GNSS, IMU, and DMI is split into several steps. The first is lab/factory precalibration, which estimates the relative offset among these sensors and their relative position to the data collecting sensors (e.g., LiDAR and cameras) [103]. The second step involves the fusion of sensor information to output the estimated positions through optimal statistical/stochastic estimators [29,104]. A typical algorithm used for this purpose is the Kalman filter (KF) [105,106,107,108], which uses continuous measurements over time with their uncertainties and a stochastic model for each sensor to estimate the unknown variables in a recursive scheme. KF is the simplest dynamic estimator that assumes linear models and Gaussian random noise of observations. As such, it is often readopted through Extended KF (EKF) for linearized nonlinear models [109,110]. However, convergence is not guaranteed for EKF, especially when the random noise does not follow the Gaussian distribution. Thus, a particle filter [111,112] is usually adopted as a good alternative, as it can simulate the noises to deal with the potential non-Gaussian noise distributions.

#### 5.2.2. Camera Calibration

Camera calibration refers to the process of rigorously determining the camera intrinsic parameters (i.e., focal length, principal point), various lens distortions (e.g., radial distortion), and other intrinsic distortions, such as affinity or decentering errors [113,114]. The camera calibration parameters often follow the standard Brown model [115] or the extended parameters [116] that additionally model in-plane errors due to film/chip displacements. The traditional and most rigorous camera calibration approach uses a 3D control field consisting of highly accurate 3D physical point arrays. Converging images of these point arrays are captured at various angles and positions. These well-distributed 3D points, together with their corresponding 2D observations on the image, go through a rigorous BA with additional parameters (i.e., calibration parameters). However, 3D control fields are demanding and costly. This approach is mostly used for calibrating survey-grade or aerial cameras at the factory level. A popular and less demanding calibration approach is called cloud-based camera calibration [116]. Instead of using the very expensive 3D control fields, this approach uses coded targets, which can be arbitrarily placed (but well-distributed with certain depth variations) in a scene as a target cloud. Converging images of these targets can yield very accurate 2D multi ray measurements, which are fed into a free-network BA for calibrating the camera parameters. Thanks to its simplicity, this is often used as a good alternative for calibrating cameras for close-range applications, including MMS applications. A less rigorous (but often used) calibration method uses a chessboard as a target for calibration [117], which extracts regularly distributed 2D measurements from images of the chessboard and performs self-calibrating BA. However, due to its limited scene coverage, the nature of the board being planar (thus lacking depth variation), and the limited flexibility in capturing well-converged images filled with features, this method may not, in BA, decorrelate camera parameters from exterior orientation parameters, leading to potential errors in calibration. Since it is very commonly used in the computer vision community and well supported by available open-source tools, it is one of the most popular approaches to obtain quick calibrations and can be used to calibrate cameras that do not demand high surveying accuracy, such as navigation cameras. 

When calibrating a multi-camera system (e.g., a stereo rig), the camera calibration is extended to additionally estimate the accurate relative orientation among these cameras. The calibration still goes through a BA, but requires at least knowledge of the scale of the targets (either the target clouds or chessboard) in order to metrically estimate the baselines between all these cameras.

#### 5.2.3. LiDAR and Camera Calibration

The calibration between LiDAR and camera covers a few aspects: first, images must be time-synchronized with the LiDAR scans. Second, the relative orientation between the LiDAR and camera rays must be computed. Third, they must have the same viewpoint to avoid parallaxes. Time-synchronization is one of the most crucial calibration steps to correct time offsets between sensors. It refers to matching the recorded and measured data from different sensors with separate clocks to create well-aligned data, in terms of time and position. Time-synchronization errors (or the time offsets) are due to (1) clock offset, which refers to the time difference between internal clocks of sensors, or (2) clock drift, which refers to sensors’ clocks operating at different frequencies or times [118]. A time offset greater than milliseconds between LiDAR and camera can cause significant positioning errors when recording an object. Additionally, the impact can be more significant and noticeable if the platform is operating at a high speed. To address this problem, sensors must have a common time reference, often based on GNSS’s time because of its high precision and ability to record positions in nanoseconds [119]. The time offset between GNSS and IMU is either neglected because of its insignificance or may be slightly corrected using KF as a fusion method. The LiDAR and camera timestamps are corrected in real-time using the computer system on board. The computer system updates that data based on GNSS’s time; some examples of these computer systems/servers include GPS service daemon (GPSD), IEEE, and Chrony.

Relative orientation refers to estimating the translation and orientation parameters between sensors. This type of calibration needs to be carried out periodically due to deteriorations of the mechanics within and among the sensors after operating in different environments. Quick calibration can be performed using a single image where four corresponding points are selected between the image and the 3D scan, using either the well-identified natural corner points or highly reflective coded targets.

Ideally, LiDAR and Camera data must be well-aligned. However, because of the wide baseline between the two sensors, they often view objects from different angles, leading to large parallaxes between them. A large parallax causes crucial distortions such as flipping or flying points, flying points, occlusions, etc. To resolve this issue, the relative orientation parameters are used to project the LiDAR data into the camera coordinate system [120].

### 5.3. Georeferencing LiDAR Scans and Camera Images Using Navigation Data

Both the LiDAR scan and images collect data in a local coordinate system. Georeferencing them refers to determining their global/geodetic coordinates, mostly based on fused GNSS/IMU/DMI positioning data. This is a process after calibration among these data collection sensors (as described in Section 5.2). Georeferencing includes the estimation of the orientation (boresight) and position (lever-arm) parameters/offsets with respect to GNSS and IMU [19]. The boresight and level-arm parameters define the geometrical relationship between positioning and data collection sensors. There are two approaches to perform georeferencing: (1) the direct approach, which uses only GNSS/IMU data, or (2) the indirect approach, which uses GNSS/IMU data in addition to GCP and BA for refinement [68]. The direct approaches are less demanding, since they do not require GCP, and they can achieve accuracy in the decimeter to centimeter levels. Indirect approaches can provide more accurate (centimeter-level) and precise results where typical surveying methods like GCPs and BA are adopted. However, they are very expensive, and their accuracy may vary based on the GCP setup (i.e., position and number of GCPs).

### 5.4. Data Processing in Preparation for Scene Understanding

Mobile mapping is highly relevant to autonomous vehicles, where scene understanding is crucial to not only automate the mapping process but also provide critical scene information in real-time to support platform mobilizations. Scene understanding is the process of identifying the semantics and geometry of objects [121,122]. With the enhanced processing capability of mobile computing units, advanced machine learning models, and the ever-increasing datasets, there is a growing trend toward performing on-board data processing and scene understanding using the collected measurements from the mobile system [123,124]. These include real-time detection, tracking, and semantic segmentation of both dynamic (e.g., pedestrians) and static (e.g., road markings or signs) objects in a scene [122,125]. This has driven the need to develop representative benchmark datasets, better-generalized training, domain adaptation approaches [126], and lighter machine learning models or network structures that support real-time result inferences [127]. Examples of these efforts include MobileNet [128], BlitzNet [127], MGNet [129], and MVLidarNet [130]. Challenges exist when addressing these needs, as the mobile platforms may collect data under extremely different illuminations (e.g., daylight and night), weather conditions (e.g., rainy, snowy, and sunny), and may utilize drastically different sensor suites with different qualities of raw data.

## 6. Applications

Mobile mapping provides valuable assets for different applications, driven by not only the broad availability of easy-to-use and portable MMS platforms but also their readiness under different operating environments. This is particularly useful as most of these applications rely on regularly acquired data for detection and monitoring purposes, such as railway-based powerline detection/monitoring [131,132]. In this section, we reviewed some of the main applications of mobile mapping technology, including road asset management, conditions assessment, BIM creation, disaster response, and heritage conservation. Documented examples of these applications in publications are shown in Table 5 and are detailed in the following subsections.

**Road Asset Management and Condition Assessment:** MMSs operating on roads can regularly collect accurate 3D data of the road and its surroundings, which facilitates road asset management, mapping, and monitoring (e.g., road signs, traffic signals, pavement dimensions) [79]. Creating road asset inventories is of great importance, given the large volume of road assets. Furthermore, since the condition of the roads deteriorates over time, automatic means of regular transportation maintenance, such as pavement crack and distress detection are critically needed [133,135]. Therefore, a key benefit of generating an updated and accurately georeferenced road asset inventory is to allow automatic and efficient change detection in place of traditionally laborious manual inspections [134].

Typically, road condition monitoring processes consist of four steps [148]: (1) data collection using MMS, (2) defect detection, which can be performed automatically using deep learning-based approaches, (3) defect assessment, and (4) road condition index calculation to classify road segments based on the type and severity of the defect. Therefore, MMS data could further assist in increasing road safety, for example by detecting road potholes [78,149], evaluating the location of speed signs before horizontal curves on roadways [150], or assessing the passing sight distance on highways [151].

**Building Information Modeling:** BIM is one of the most well-established technologies in the industry of architecture, engineering, and construction. It provides an integrated digital database about an asset (e.g., building, tunnel, bridge, or 3D model of a city) during the project’s life cycle. Typical BIM stages include (1) rigorous data collection, preparation of the 2D plans, and upload of these into specialized software programs to convert them into a digital format. The collected data include information such as architectural design (i.e., materials and dimensions), structural design (e.g., beams, columns, etc.), electrical and mechanical designs, sewage systems, etc.; (2) preparation of the 2D plans; and (3) manual upload and update of the plans using specialized software. This can facilitate the design, maintenance, and renovation processes of engineering buildings and infrastructures. However, this can be a challenging task because of the amount of data that need to be collected and the lack of automated processes that can increase the time and cost.

Nowadays, MMSs have been widely adopted for BIM projects due to their high accuracy, time efficiency, and lower cost in collecting 3D data. The collected point clouds and images are used to produce the 3D reconstructed model of an asset, then processed under semantic segmentation or classification to extract detailed information of all elements in the asset. The final product is then transferred to the BIM software to extract and simulate important information related to the life cycle of the project. In general, MMS can provide sufficiently accurate results for the derived BIM products [20]. These derived products can be either 2D floor plans or 3D mesh or polyhedral models representing the structure of architecture or the life cycle of the construction process [145]. A popular example of MMS in BIM is 3D city modeling, where MMS can be used to collect information on roadside buildings [143,144,145] and their structural information (e.g., window layout and doors) [98,146]. Additionally, they can also be used to maintain plans and record indoor 3D assets and building layouts, which can be generated using handheld, backpack, or trolley MMSs.

**Emergency and Disaster Response:** The geospatial data provided by the MMSs are critical to improving emergency, disaster responses, and post-disaster recovery projects. MMSs provide cost and time-efficient solutions to collect and produce 3D reconstructed models with detailed information about the semantics and geometry to navigate through an emergency or a disaster. MMSs have been reported to provide building-level information (e.g., floors, walls, and doors) to a resolution/accuracy at the centimeter level. In many cases, the building’s plans are not up-to-date after construction, which may hinder a rescue mission in case of a fire emergency [139,152]. On the other hand, the MMS can provide an efficient alternative to produce an accurate and updated 3D model of a facility or a building at a minimal cost, which facilitates emergency responses. Another example is collecting 3D data on roadside assets and feeding them into GIS systems, which can serve as pre-event analysis tools to identify potential impacts of natural disasters through simulation (e.g., flood simulation, earthquake, etc.), aiding preventative planning. The future directions of MMSs involve more efficient data collection methods (as simple as a man holding a cellphone imaging surroundings), and although these might involve lower accuracy [3], they could supply critical georeferenced information in a disastrous or emergency event to supply information for situation awareness and remedies.

**Vegetation Mapping and Detection:** Mobile mapping has shown great success in collecting high-resolution and detailed vegetation plots, used to create up-to-date digital tree inventories for urban green planning and management. This has greatly accelerated traditionally laborious visual inspections [142]. In addition, vegetation monitoring is important to limit declines in biodiversity and identify hazardous trees [153,154]. Therefore, these requirements impacted the advancement of keeping an up-to-date digital database for vegetation data. The collected 3D data could be used to model 3D trees for visualization purposes in urban 3D models. Typically, the workflow might consist of three main steps [155]: (1) tree detection by segmenting the generated point clouds, (2) simplifying the detected structure of point clouds, (3) deriving the geometry parameters, such as canopy height and crown width and diameter [155,156]. Then, the collected point cloud could be used to detect trees and low vegetation at the roadside [141,143] to take into account the occlusion on building facades and supply information for city modeling. Moreover, the collected data from MMSs could also be used in calculating urban biomass, combatting the urban heat island effect, and helping in analyzing the influence of the ecosystem on climate change [157].

**Digital Heritage Conservation:** There is a growing trend toward people realizing the importance of digitally documenting archaeological sites and preserving cultural heritage [146,158,159]. Many of these sites are in danger of deterioration and collapse, which may be accelerated due to extreme weather and natural disasters, such as the collapse of many cultural sites in Nepal and Iran due to earthquakes [160]. Therefore, there is a critical need to proactively document these sites while they are still in shape [92,161,162]. Moreover, a well-documented heritage site may enable other means of tourism, such as virtual tours, to off-load site visitation and reduce the human factors that impact the deterioration of these sites. As a means of collecting highly accurate 3D data, MMS has been used as one of the primary sources to create 3D models of complex and large archaeological heritage sites. The data collection process for these sites often requires multi-scans of both the interior and exterior from different angles to generate occlusion-free, realistic 3D models. For example, a vehicle-mounted system could be used to drive around the sites to collect exterior information, and wearable/handheld devices used to scan their interiors [162].

### Summary

We discussed selected applications of mobile mapping that demonstrate the importance and necessity of utilizing MMSs in different scenarios. The adoption of mobile mapping technology in various applications has been proven to not only increase productivity but also reduce the cost of operation. For instance, using digital assets for construction has led to a boost in productivity for the global construction sector by 14–15% [163]. In addition, digitizing historic structures through creating BIMs using mobile mapping data will enable preventive conservation for heritage buildings, saving 40–70% on maintenance costs [164]. Aside from the productivity and cost aspects, mobile mapping data pave the way for producing new road monitoring studies and methods that will increase road safety and dramatically reduce the probability of accidents [165].

## 7. Conclusions

### 7.1. Summary

In this paper, we provided a thorough review of state-of-the-art mobile mapping systems, their sensors, and relevant applications. We reviewed sensors and sensor suites typically used in modern MMSs and discussed, in detail, their types, benefits, and limitations (Section 3). Then, we reviewed mobile platforms, including vehicle-mounted, handheld, wearables, etc., and described, in detail, their collection logistics, giving examples of modern systems of different types (Section 4). We also specifically highlighted their supported-use case scenarios. We further reviewed the critical processing steps that turn raw data into the final mapping products (Section 5), including sensor calibration, fusion, and georeferencing. Finally, we summarized the most common applications (Section 6) that currently utilize the capabilities of modern MMSs.

### 7.2. Future Trends

Despite the many variations of MMSs and their sensor suites, the main goal of an MMS is to provide a means of collecting 3D data at close range, with maximal flexibility and minimal cost. Given the complex terrain environment, a single MMS or even a few MMSs could hardly be sufficient at all levels of mobile mapping applications. Thus, while off-the-shelf solutions are partially available, developing or adapting MMSs to designated applications is an ongoing effort. To date, MMS is still regarded as an expensive collection means, as the equipment, sensors, and manpower required to handle the logistics and processing are still considerable. Therefore, as far as we can conclude, ongoing and future trends continue to be:

(1) Reduced sensor cost for high-resolution sensors, primarily LiDAR systems with equivalent accuracy/resolution as those currently in use, but at a much lower cost.
(2) Crowdsourced and collaborative MMS using smartphone data; for example, the new iPhone has been equipped with a low-cost LiDAR sensor.
(3) Incorporation of new sensors, such as ultra-wide-band tracking systems, as well as WiFi-based localization for use in MMS.
(4) Enhanced (more robust) use of cameras as visual sensors for navigation.
(5) Higher flexibility in sensor integration and customization as well as more mature software ecosystems (e.g., self-calibration algorithms among multiple sensors) to allow users to easily plug and play different sensors to match the demand for mapping different environments.
(6) Advanced post-processing algorithms for pose estimation, data registration for a close-range scenario, dynamic object removal for data cleaning, and refinement for collections in cluttered environments.
(7) The integration of novel deep learning solutions at all levels of processing, from navigation and device calibration to 3D scene reconstruction and interpretation.

Given the complexity of MMSs and their application scenarios, a one-stop-shop solution arguably does not exist. However, it could be possible to streamline and optimize the customization of a system if the above-mentioned challenges were consistently attacked. Our future work will encompass component-level surveys that provide the community with comprehensive views accelerating the convergence of solutions addressing the above-mentioned efforts.

## Figures and Tables

**Table 1:** Positioning sensors overview.

Sensor	Description	Benefits	Limitations
GNSS receiver	The signals from orbiting satellites are utilized by the GNSS receiver to compute the position, velocity, and elevation. Some examples include GPS, GLONASS, Galileo, and BeiDou.	No/less accumulation of errors due to its dependence on external signals. Data collected under a global reference coordinate system (e.g., WGS84).	Signal inaccessible in complex urban regions e.g., tall buildings, trees, tunnels, indoor environments, etc. Requires post-processing using DGPS and RTK-GPS to minimize errors from receiver’s noise, pseudo-range, carrier phase, doppler shifts, atmospheric delays, etc.
IMU	IMU is an egocentric sensor that records the relative position of the orientation and directional acceleration of the host platform.	Capable of navigating in all environments, such as indoors, outdoors, tunnels, caves, etc. A necessary supplemental data source for urban environments where GPS is unstable.	Requires consistent calibration and a reference to avoid drift from the true position. Limited to short-range navigation.
DMI	A supplementary positioning sensor measures the traveled distance of the platform, i.e., information derived from a speedometer.	A supplemental sensor to provide additional data points to alleviate accumulation errors of IMU sensors.	Requires calibration and provides only distance information (1 degree of freedom).

**Table 2:** Specifications of different LiDAR sensors.

	Company	Model	Range (m)	Range Accuracy (cm)	Number of Beams	Horizontal FoV (°)	Vertical FoV (°)	Horizontal Resolution (°)	Vertical Resolution (°)	Points Per Second	Refresh Rate (Hz)
**Rotating**	RIGEL	VQ-250	1.5–500	0.1	—	360	—	—	—	300,000	—
		VQ-450	1.5–800	0.8	—	360	—	—	—	550,000	—
	Trimble	MX50 laser scanner	0.6–80	0.2	—	360	—	—	—	960,000	—
		MX9 laser scanner	1.2–420	0.5	—	360	—	—	—	1,000,000	—
	Velodyne	HDL-64E	120	±2	64	360	26.9	0.08 to 0.35	0.4	1,300,000	5 to 20
		HDL-32E	100	±2	32	360	41.33	0.08 to 0.33	1.33	695,000	5 to 20
		Puck	100	±3	16	360	30	0.1 to 0.4	2.0	300,000	5 to 20
		Puck LITE	100	±3	16	360	30	0.1 to 0.4	2.0	300,000	5 to 20
		Puck Hi-Res	100	±3	16	360	20	0.1 to 0.4	1.33	300,000	5 to 20
		Puck 32MR	120	±3	32	360	40	0.1 to 0.4	0.33 (min)	600,000	5 to 20
		Ultra Puck	200	±3	32	360	40	0.1 to 0.4	0.33 (min)	600,000	5 to 20
		Alpha Prime	245	±3	128	360	40	0.1 to 0.4	0.11 (min)	2,400,000	5 to 20
	Ouster	OS2-32	1 to 240	±2.5 to ±8	32	360	22.5	0.18	0.7	655,000	10, 20
		OS2-64	1 to 240	±2.5 to ±8	64	360	22.5	0.18	0.36	1,311,000	10, 20
		OS2-128	1 to 240	±2.5 to ±8	128	360	22.5	0.18	0.18	2,621,000	10–20
	Hesai	PandarQT	0.1 to 60	±3	64	360	104.2	0.6°	1.45	384,000	10
		PandarXT	0.05 to 120	±1	32	360	31	0.09, 0.18, 0.36	1	640,000	5, 10, 20
		Oandar40M	0.3 to 120	±5 to ±2	40	360	40	0.2, 0.4	1, 2, 3, 4, 5, 6	720,000	10, 20
		Oandar64	0.3 to 200	±5 to ±2	64	360	40	0.2, 0.4	1, 2, 3, 4, 5, 6	1,152,000	10, 20
		Pandar128E3X	0.3 to 200	±8 to ±2	128	360	40	0.1, 0.2, 0.4	0.125, 0.5, 1	3,456,000	10, 20
**Solid-state**	Luminar	IRIS	Up to 600	—	640 lines/s	120	0–26	0.05	0.05	300 points/square degree	1 to 30
	Innoviz	InnovizOne	250	—	—	115	25	0.1	0.1	—	5 to 20
		InnovizTwo	300	—	8000 lines/s	125	40	0.07	0.05	—	10 to 20
**Flash**	LeddarTech	Pixell	Up to 56	±3	—	117.5 ± 2.5	16.0 ± 0.5	—	—	—	20
	Continental	HFL110	50	—	—	120	30	—	—	—	25

“—” indicates that the specifications were not mentioned in the product datasheet.

**Table 3:** Camera sensor overview.

Type	Description	Benefits	Limitations
Monocular	Single-lens camera.	Low cost. Provides a series of single RGB images to collect high-resolution and geotagged images or panoramas.	Cannot recover 3D scale without additional sensors. Camera networks suboptimal to generate highly accurate 3D points.
Binocular	Two collocated cameras with known relative orientation capturing overlapping and synchronized image	Can provide depth and scale of objects the scene. Provides better accuracy integrated with LiDAR sensor.	Performance and accuracy may depend on the algorithm used to compute the 3D information.
RGB-D	Cameras that capture RGB and depth images at the same time	Simultaneous data acquisition. Provides high accuracy when integrated with LiDAR.	Depth image sensitive to occlusions. Low range. The depth image may include some uncertainties and errors.
Multi-camera system	A spherical camera system with multiple cameras that can provide a 360° field of view	Panoramic view showing the entire scene. Suitable for street mapping applications.	Requires large storage to save images in real-time. Must be properly calibrated to assure alignment of images and minimum distortions.
Fisheye	Spherical lens camera that has more than 180° field of view	Provides wide coverage of the scene allowing capture of the scene with fewer images.	Lens distortions. Non-projective transformation. Requires rigorous calibration.

**Table 4:** Specifications of different MMSs.

	System	Release Year	Indoor	Outdoor	Camera	LiDAR/Max. Range	IMU	GPS	Accuracy *	Applications
Vehicle-mounted	Leica Pegasus: Two Ultimate	2018	🗶	🗸	360° FoV	ZF9012 profiler 360° × 41.33°/100 m	🗸	🗸	2 cm horizontal accuracy 1.5 cm vertical accuracy	Urban 3D modeling. Road asset management. Analyzing change detection Creating HD maps. Generating geolocated panoramic images.
	Teledyne Optech Lynx HS600-D	2017	🗶	🗸	360° FoV	2 Optech sensors/130 m	🗸	🗸	±5 cm absolute accuracy	
	Topcon IP-S3 HD1	2015	🗶	🗸	360° FoV	Velodyne HDL-32E LiDAR/100 m	🗸	🗸	0.1 cm road surface accuracy (1 sigma)	
	Hi-Target HiScan-C	2017	🗶	🗸	360° FoV	650 m	🗸	🗸	5 cm at 40 m range	
	Trimble MX7		🗶	🗸	360° FoV	🗶	🗸	🗸	—	
	Trimble MX50	2021	🗶	🗸	90% of a full sphere	2 MX50 Laser scanner/80 m	🗸	🗸	0.2 cm (laser scanner)	
	Trimble MX9	2018	🗶	🗸	1 spherical + 2 side looking + 1 backward/downward camera	MX9 Laser scanner/up to 420 m	🗸	🗸	0.5 cm (laser scanner)	
	Viametris vMS3D	2016	🗶	🗸	FLIR Ladybug5+	Velodyne VLP-16 + Velodyne HDL-32E	🗸	🗸	2–3 cm relative accuracy	
Handheld	HERON LITE Color	2018	🗸	🗸	360° × 360° FoV	1 Velodyne Puck/100 m	🗸	🗶	3 cm relative accuracy	Mapping enclosed and complex spaces and cultural heritage. Forest surveying. Building Information Modeling.
	GeoSLAM Zeb Go	2020	🗸	🗶	Can be added, accessory	Hokuyo UTM-30LX laser scanner/30m	🗶	🗶	1 to 3 cm relative accuracy	
	GeoSLAM Zeb Revo RT	2015	🗸	🗶	Can be added, accessory	Hokuyo UTM-30LX laser scanner/30m	🗶	🗶	0.6 cm relative accuracy	
	GeoSLAM Zeb Horizon	2018	🗸	🗸	Can be added, accessory	Velodyne Puck VLP-16/100 m	🗶	🗶	0.6 cm relative accuracy	
	Leica BLK2GO	2018	🗸	🗸	3 camera system 300° × 150° FoV	Up to 25 m 360 × 270	🗶	🗶	±1 cm in an indoor environment with a scan duration of 2 min	
Wearable	Leica Pegasus: Backpack	2017	🗸	🗸	360° × 200° FoV	Dual Velodyne VLP-16/100 m	🗸	🗸	2 to 3 cm relative accuracy 5 cm absolute accuracy	
	HERON MS Twin	2020	🗸	🗸	360° × 360° FoV	Dual Velodyne Puck/ 100 m	🗸	🗶	3 cm relative accuracy	
	NavVis VLX	2021	🗸	🗸	360° FoV	Dual Velodyne Puck LITE/100 m	🗸	🗶	0.6 cm absolute accuracy at 68% confidence 1.5 cm absolute accuracy at 95% confidence	
	Viametris BMS3D-HD	2019	🗸	🗸	FLIR Ladybug5+	16 beams LiDAR + 32 beams LiDAR	🗸	🗸	2 cm relative accuracy	
Trolley	NavVis M6	2018	🗸	🗶	360° FoV	6 Velodyne Puck LITE/100 m	🗸	🗶	0.57 cm absolute accuracy at 68% confidence 1.38 cm absolute accuracy at 95% confidence	Indoor mapping for government buildings, airports, and train stations. Tunnel inspection. Measuring asphalt roughness. Building Information Modeling.
	Leica ProScan	2017	🗸	🗸	🗶	Leica ScanStation P40, P30 or P16	🗸	🗸	0.12 cm (range accuracy for Lecia ScanStation P40)	
	Trimble Indoor	2015	🗸	🗶	360° FoV	Trimble TX-5, FARO Focus X-130, X-330, S-70-A, S-150-A, S-350-A	🗸	🗶	1 cm relative accuracy when combined with FARO Focus X-130	
	FARO Focus Swift	2020	🗸	🗶	HDR camera	FARO Focus Laser Scanner with a FARO ScanPlan 2D mapper	🗸	🗶	0.2 cm relative accuracy at 10 m range 0.1 cm absolute accuracy	

* The accuracy measurement reported by the manufacturers. The measure of the accuracy is unknown if not stated as relative or absolute. The “—” symbol indicates that the specifications were not mentioned in the product datasheet.

**Table 5:** An overview of the selected mobile mapping applications.

	Selected Applications	Highlights
Road asset management and condition assessment	Extraction of road assets [79]; road condition assessment [133]; detection of pavement distress using deep-learning [134]; evaluation of pavement surface distress for maintenance planning [135].	Vehicle-mounted system regularly operating on the road. More efficient than manual inspection. Leveraging deep learning to facilitate the inspection process.
BIM	Low-cost MMS for BIM of archeological reconstruction [136]; analysis of BIM for transportation infrastructure [137].	Data are collected with portable systems. Useful for maintenance and renovation planning. Rich database for better information management.
Emergency and disaster response	Network-based GIS for disaster response [138]; analyzing post-disaster damage [139].	Timely and accurate disaster response. Facilitates the decision-making process. Effective training and simulations.
Vegetation mapping and detection	Mapping and monitoring riverine vegetation [140]; tree detection and measurement [141,142,143].	Accurate and automatic measurements. Reduces occlusions for 3D urban models.
Digital Heritage Conservation	Mapping a complex heritage site using handheld MMS [92]; mapping a museum in a complex building [94]; numerical simulations for structural analysis of historical constructions [144]; digital heritage documentation [145]; mapping archaeological sites [146]; development of a digital heritage inventory system [147].	Utilizes the flexibility of portable platforms. Enables virtual tourism. Digital recording of cultural sites.

## Data Availability

Not applicable.
